# Dynamics of Polymeric Re-Entrant Auxetic Structures: Cyclic Compression Studies

**DOI:** 10.3390/polym17060825

**Published:** 2025-03-20

**Authors:** Julian Plewa, Małgorzata Płońska, Grzegorz Junak

**Affiliations:** 1 Faculty of Science and Technology, Institute of Materials Engineering, University of Silesia in Katowice, 75 Pułku Piechoty Str., 41-500 Chorzów, Poland; malgorzata.plonska@us.edu.pl; 2 Faculty of Materials Engineering, Silesian University of Technology, 8 Krasinskiego Str., 40-019 Katowice, Poland; grzegorz.junak@ps.edu.pl

**Keywords:** auxetic structures, re-entrant unit cells, hysteresis, Mullins effect

## Abstract

The present study investigated the dynamic behavior of structures made of re-entrant unit cells subjected to cyclic compressive loading limited to the elastic range. The structures were assembled from printed polymer re-entrant cells in six combinations. Through the given compression cycles for three different amplitude values, strain-force relationships, which had the shape of a hysteresis loop, were obtained. Under compression, all unit cells of the structures deformed uniformly, though only for a certain amount of strain, whereas with larger changes, they underwent uncontrolled deformation. Experiments showed that structures composed of more than one unit cell exhibit different mechanical characteristics. It was observed that the width of the hysteresis loop depended on the degree of closing the structure and on the compression amplitude. The obtained hysteresis curves for different amplitudes also testify to the occurrence of the Mullins effect for these polymeric auxetic structures. Taking into account the maximum values of changes in dimensions for a given compression cycle, Poisson’s ratio values were determined, which were negative and below unity. The effect of strut thickness on the NPR was confirmed, decreasing its negative value along with the increasing thickness.

## 1. Introduction

Auxetic structures, which constitute a subset of mechanical metamaterials, are an object of considerable interest in science and technology. This is due to their unique way of deformation, expressed by negative Poisson’s ratio (NPR). The emergence of NPR stands in contrast to the everyday experience of stretching objects, which become thinner as they lengthen and thus have a positive Poisson’s ratio. Auxetic structures, on the other hand, become wider when expanding and thinner when contracting. Poisson’s ratio ν is one of the most important indicators of the properties of auxetic structures, defined as the negative ratio of transverse and longitudinal strain. Yet, to produce an auxetic structure, a special arrangement of unit cells and free spaces between them is needed. Such structures have unit cells arranged regularly and connected to each other—usually with flexible joints. This mainly involves structures with elastic properties [1].

**Figure 1:**
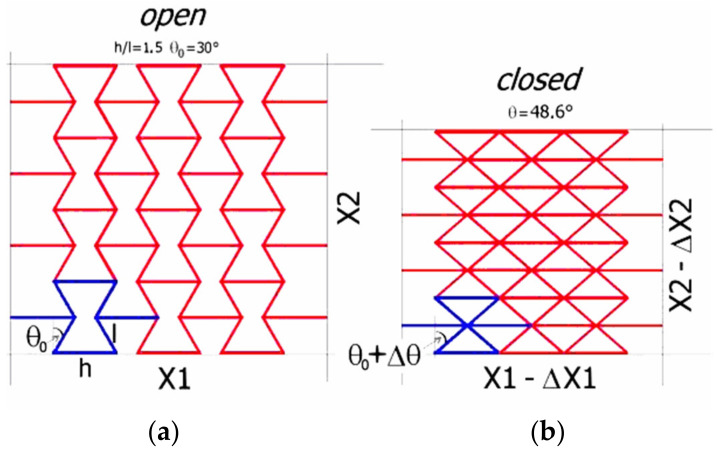
Theoretical re-entrant structure (5 × 4) in an *open* (**a**) and *closed* (**b**) position with the geometric parameters h, l, and θ, as well as the linear dimensions.

However, it is also possible to theoretically design and practically implement other auxetic structures, i.e., rigid and inelastic ones, with hinged unit cells [2,3]. The numerous potential advantages of auxetic structures that have been demonstrated theoretically due to the combination of lightness and high strength resulted in a wide range of proposed applications, e.g., in protection systems [4,5,6,7,8], sensors [4,5,9,10], machinery and transportation [4,5,11,12], biomedicine [4,5,13,14,15], and sporting equipment [4,5,16,17]. These aspects of application have received particularly extensive treatment in numerous review publications, the number of which has exceeded one hundred publications since 2000. Given the above, it would be interesting to see the extent to which these theoretical speculations have been implemented in practice and the benefits they brought. Exploring this question requires staying up to date with the new technical advances and patent solutions in this area. Auxetic structures made of re-entrant unit cells are derived from Almgren [18], who first proposed arrangements of rigid rods joined by elastic hinges in the form of computer graphics. The basic re-entrant cell is a hexagon with four acute angles. It is made up of struts: a horizontal one h and a diagonal one l, and the theta angle, defined as in Figure 1. A structure composed of connected unit cells subjected to a compressive load changes its dimensions vertically and horizontally. This change in dimensions is due, in the first place, to the bending of the strut joints, which is expressed by a change in theta angle. Strut joints must exhibit elastic properties, which enables them to undergo dimensional changes under load. The magnitude of the load is adjusted for the range of dimensional changes. As the load increases, the struts may also deform (bend) themselves, first elastically and then plastically.

In theoretical studies, individual re-entrant cells subjected to compression contract horizontally and vertically. Many studies of this kind have simulated this unit cell behavior, as discussed in a recent review paper [19]. These vast numbers of simulation studies only examine virtual re-entrant structures in which entire unit cells contract in compression and expand in tension. Although such behavior of re-entrant cells also generally occurs in practice, in some cases, an opposite phenomenon has been identified, i.e., the vertical expansion of re-entrant cells in compression. One work [20] described a non-auxetic deformation of a re-entrant unit cell with the horizontal struts deforming into a w-shape.

Simulation studies determine the relationships between elastic measures and the geometry of the re-entrant unit cell—as summarized in reviews [1,19]. Such theoretical studies mainly involve considering mathematical relationships in elastic materials, yielding theoretical relationships between material moduli, Poisson’s ratio, and geometric parameters of the re-entrant cell.

In contrast, experimental studies refer to actual metamaterials, for which the functional relationships relate to both the initial state of the structure and the present degree of deformation. For real structures, the Poisson’s ratio depends not only on unit cell geometry but also on the initial and instantaneous degree of deformation, expressed by the values of θ_0_ and Δθ, i.e., the initial angle between the struts as well as its change.

The present work addresses the deformation of structures at which they retain their shape—corresponding to the range of elastic deformation. Through experimental and analytical studies, the deformation behavior of re-entrant auxetic structures was analyzed for a given re-entrant angle value. The results of the experiments are presented in the form of hysteresis loops for structures tested under compressive conditions. Thus, the present work aims to observe and analyze the behavior of physical re-entrant structures with elastic properties subjected to cyclic compression.

## 2. Theoretical Analysis of a Re-Entrant Structure

Auxetic structures, in their basic form, are an ordered arrangement of interconnected unit cells with free spaces between them. Thus, in the case of a re-entrant structure, it is an arrangement of rods that act as struts.

A schematic of the structure built with re-entrant unit cells is shown in Figure 1.

The transition from the *open* system, defined by the initial angle θ_0_, to the *closed* system corresponds to the dynamic change in the structure’s dimensions.

The relative change in linear dimensions of the structure is given by the following formulae [20]:

(1) $$\frac{\Delta X1}{X1}=\frac{\sin\theta_{0}-\sin(\theta_{0}\pm \Delta \theta )}{\frac{2n-1}{2(n-1)}\frac{h}{l}-\sin\theta_{0}},$$

(2) $$\frac{\Delta X2}{X2}=\frac{\cos(\theta_{0}\pm \Delta \theta )-\cos\theta_{0}}{\cos\theta_{0}},$$

where θ_0_ is the initial angle (re-entrant angle), Δθ is the change in the angle due to deformation, with the + sign referring to compression and the − sign referring to the tension of the structure, h is the length of the horizontal strut, l is the length of the diagonal strut, and n is the number of unit cells horizontally. These theoretical formulae describe the dynamics of the movement of structures expressed by the change in the angle θ. The formulae (1) do not capture either the magnitude of the load needed to induce dimensional changes or the material constants of the unit cell material. Moreover, theoretical formulae of this type do not take into account the thickness or the width of the struts. They can thus be treated as universal relationships that can correspond to physical conditions provided that the following requirements are met:

1. The struts are not bent;
2. Deformation occurs due to the elastic bending of strut joints;
3. Deformation can only occur in the range in which:

- h/l < 2, the θ_limit_ angle given by the following formula:
  (3) $$\frac{h}{2l}=sin(\theta_{limit})$$
  is not exceeded;
- the h/l > 2 limit angle is smaller than the one given by the inequality θ_limit_ < 90°.

The above geometric relationships (1) are sufficient to determine the Poisson’s ratio.

(4) $$\nu_{12}=-\frac{\frac{\Delta X1}{X1}}{\frac{\Delta X2}{X2}},\;\nu_{21}=-\frac{\frac{\Delta X2}{X2}}{\frac{\Delta X1}{X1}}$$

Based on Equations (1) and (4), it is possible to analyze how the value of Poisson’s ratio for re-entrant structures changes as a function of angle changes Δθ for the assumed geometric parameters h/l, θ_0_. If one relates such analyses to physical conditions, it is easier to choose the measurable relative change in linear dimensions ΔX1/X1 as an independent variable instead of Δθ.

The following shows how the Poisson’s ratio changes for re-entrant structures under compression. By selecting re-entrant unit cells (i.e., h/l) for the auxetic structure, the required Poisson’s ratio values can be obtained by assigning an initial angle of θ_0_ (entry angle). When choosing θ_0_, however, one must take into account the condition (4), and for example, for h/l = 0.5, the angle must be less than 14.8°, while for h/l > 2, a wider range of less than 90° is available.

First of all, a general relationship for re-entrant structures is visible, namely, that Poisson’s ratio widely varies with the level of strain. As the strain increases, the negative value of the Poisson’s ratio decreases.

**Figure 2:**
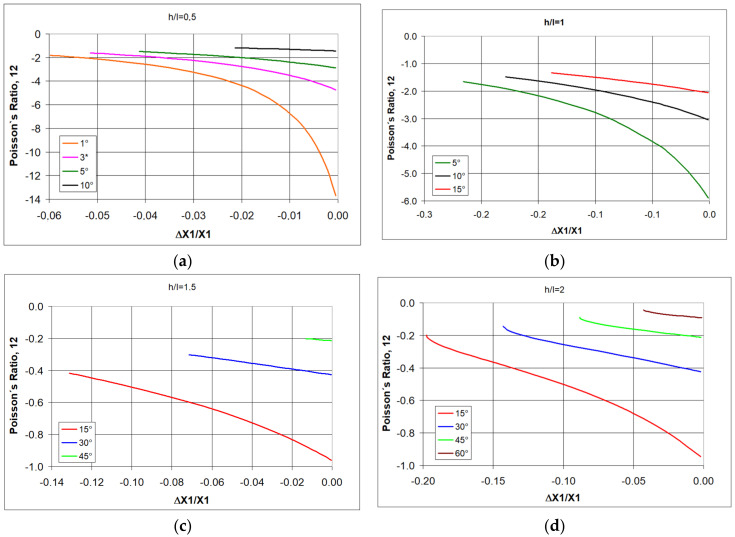
The value of Poisso’s ratio as a function of relative contraction when compressing structures of re-entrant cells with parameters h/l = 0.5, (**a**) h/l = 1, (**b**) h/l = 1.5 (**c**), and h/l = 2 (**d**), for given values of θ.

It is also strongly affected by the change in the h/l ratio. For short re-entrant unit cells, very large negative Poisson’s ratio-NPR values are obtained (Figure 2a), bearing in mind that for relatively large ν_12_, its inverse ν_21_ is very small.

As the h/l ratio increases, i.e., as the re-entrant unit cells lengthen, the NPR values decrease (Figure 2). In addition to these properties, for the analyzed structures, there is a clear effect of the degree of compressive strain on the NPR value. As the strain (relative contraction) increases, negative Poisson’s ratio (NPR) decreases.

**Figure 3:**
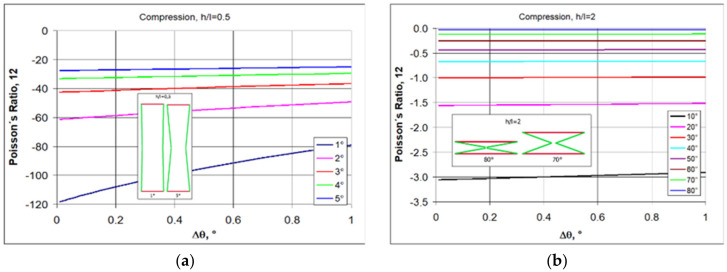
Poisson’s ratio as a function of angle change Δθ for the given values of θ_0_, re-entrant unit cells with h/l = 0.5 (**a**) and h/l = 2 (**b**).

These differences are clearly visible for the dependence of the Poisson’s ratio on the change in angle Δθ in compression. While the change in angle Δθ corresponds to the elastic bending of the strut joints, it is often small. The elastic dimensional change in materials is, after all, also small. Figure 3 shows the change in Poisson’s ratio as a function of Δθ for small changes in this angle.

For elongated re-entrant unit cells (Figure 3a), these small angle changes in compression correspond to very large NPR. Meanwhile, for wide unit cells (h/l ≥ 2), NPR is much smaller and almost constant for this range of angle changes (Figure 3b).

In this case, one can consider the Poisson’s ratio to be constant. The effect of the geometry of the re-entrant unit cells (i.e., h/l) on the auxetic properties of the structures can be illustrated for a selected value of the initial angle θ_0_.

**Figure 4:**
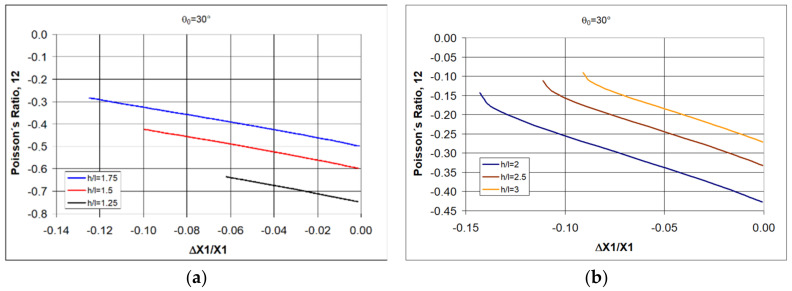
Relationship between Poisson’s ratio and the amount of relative contraction for structures of re-entrant cells with an initial angle θ_0_ = 30° for given values of h/l < 2 (**a**) and h/l > 2 (**b**).

Poisson’s ratio values for structures with an angle θ_0_ = 30° obtained in compression fall in the range −1 < ν_12_ < 0 (Figure 4).

The presented relationships indicate that with increasing h/l, the negative value of the Poisson’s ratio decreases, whereas with decreasing h/l, the Poisson’s ratio increases. This trend was also highlighted in the paper [21].

**Figure 5:**
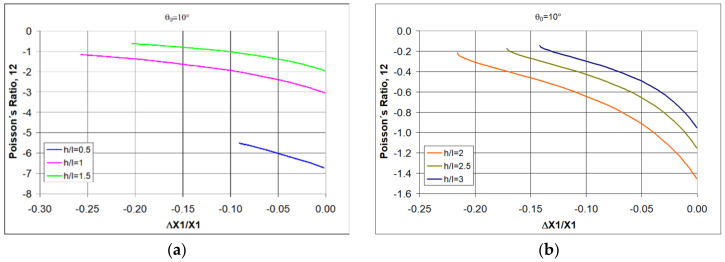
Relationship between Poisson’s ratio and the amount of relative contraction for structures of re-entrant cells with an initial angle θ_0_ = 10° for given values of h/l < 2 (**a**) and h/l > 2 (**b**).

For higher NPR values, it is necessary to use re-entrant unit cells with a smaller initial angle value θ_0_. Figure 5 shows the relationships obtained for the angle θ_0_ = 10°.

The results of the theoretical calculations shown in Figure 5 indicate that in order to obtain high values of Poisson’s ratio, it is necessary to use re-entrant unit cells with reduced values of both θ_0_, as well as selecting the particularly favorable cell geometry with h/l < 1—Figure 5a.

Summarizing these theoretical considerations, it can be pointed out that the proper functioning of such structures is due to the combination of rigid and flexible elements—the struts remain rigid, whereas their connections become flexible. Rigid elements are used to transmit the load, while flexible elements enable movement. These principles, however, cannot be fully implemented in physical structures since both the rigid elements (struts) and their joints (bends) are made of the same material—preferably a semi-rigid one. This limits the potential for large dimensional changes in the elastic range. Furthermore, there is an additional parameter of thickness to consider that affects the rigidity of the struts and their joints. It is known in practice that the geometric dimensions of an element affect its stiffness, i.e., increasing the thickness of a strut increases its stiffness and thus changes its deformation behavior. This parameter is not accounted for in the theoretical Equations (1)–(4).

The presented theoretical analysis utilizing relations (1) and (4), related to the projected movement of the structure, provides a starting point for building physical structures and observing the dynamics of their compression. In this case, using the semi-rigid unit cell material, it is possible to seek answers to open questions about the nature and quality of deformation of the structures, depending on their complexity and deformation type. The answers to such questions are sought in the experiments described in the next section.

## 3. The Experiments

This section reports the results of compression tests on spatial structures formed from symmetrically distributed connected re-entrant cells.

Flat and spatial metamaterial structures were made using an incremental method (3D Ender printer, FIBERFLEX 400 Filament, Fiberlab S.A., Brzezie, Poland). The filament parameters are as follows: E = 4 GPa, ε = 6%, σ = 37 MPa, T_g_ = 60 °C. The design principle of the structures under consideration was increasing the number of unit cells, and the main objective was to study the effect of expanding the structure on its observed mechanical characteristics.

**Figure 6:**
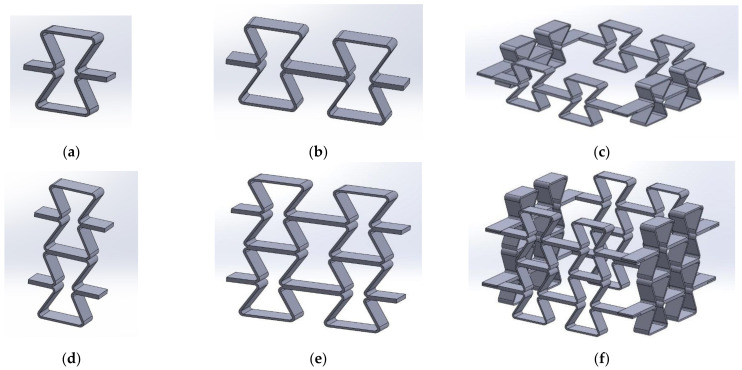
(**a**–**f**) Schematics of structures made of re-entrant cells with dimensions h = 46 mm, l = 32.6 mm *, θ_0_ = 30° (thickness t = 2.1–2.4 mm, width s = 15 mm). * This value was for the diagonal strut without curves (for which the θ_limit_ = 44.8°), which corresponded to a theoretical value of 37.2 mm (for which the θ_limit_ = 38.2°).

Schematics of the manufactured structures are shown in Figure 6.

The curved joints used helped avoid the problem of sharp corners with destructive stresses piling up.

The compression experiment consisted of typical cyclic compression and relaxation of structures in 5 cycles at an assumed amplitude using a Material Testing B2.5 testing machine, Zwick (Ulm, Germany), with automatic registration of compressive force and deformation. For the maximum vertical deformation (amplitude), the width of the compressed structure was measured. These values were used to calculate Poisson’s ratio.

Compression testing of the structures was non-destructive, with the vertical dimensional changes for a single re-entrant cell being smaller than those given by the following formula:

ΔX2 < 2l(−sin θ_0_ + sin θ_limit_), (5)

which corresponds to the value ΔX2 = 15.1 mm (for θ_limit_ = 44.8°).

It was observed that for an amplitude of ΔX2 ≤ 10 mm, the tested structures behaved as homogeneous elastic media. This demonstrated the elastic behavior of structures under cyclic compression. The change in dimensions resulted from bending the connections between the horizontal and diagonal struts. All of the tested structures maintained a regular shape during deformation but only up to 10 mm, whereas for ΔX2 ≥ 10 mm, buckling and non-regular shape changes occurred.

## 4. Compression Tests of Re-Entrant Structures

The manufactured re-entrant structures were inspected using a testing machine with a 10 mm/min compression rate, amplitudes of 5, 7, and 10 mm, with five repetitions per cycle.

**Figure 7:**
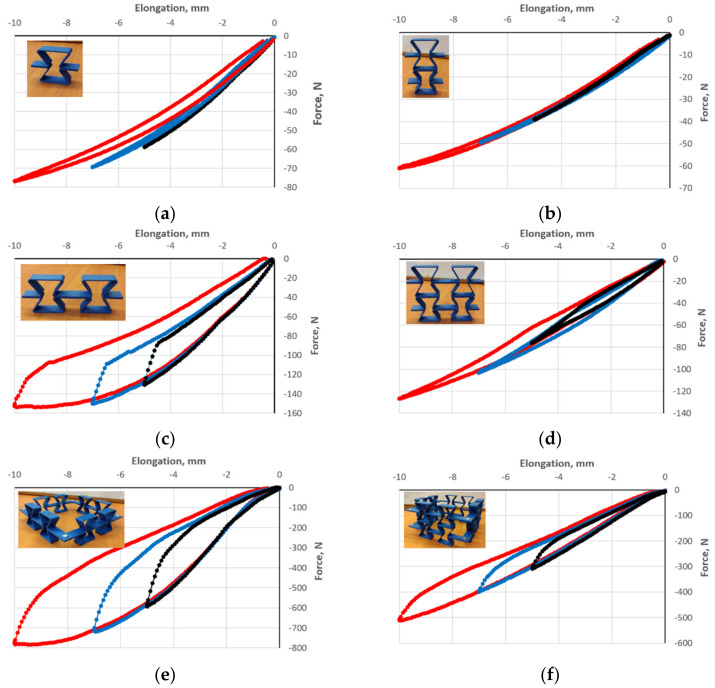
Experimental compression curves for structures of re-entrant cells with dimensions h = 46 mm, l = 32.6 mm, θ_0_ = 30° (thickness t = 2.1–2.4 mm, width s = 15 mm) with photographs, for structures (**a**–**f**) shown schematically in Figure 6. The amplitude is indicated by color: black 5 mm, blue 7 mm, and red 10 mm.

Figure 7 shows strain-force response curves for three preset strain values in the vertical direction. The results indicate that the geometry and connections of unit cells significantly affect their mechanical properties and deformation behavior.

The polymer structures were subjected to five cycles of loading and release, performed for amplitudes of 5, 7, and 10 mm marked with black, orange, and green, respectively (Figure 7). The obtained strain-force curves coincide for a given amplitude, except for the curve for the first compression cycle, which is below for structures at Figure 7a–c—corresponding to a higher value of the compressive load. In contrast, for two-layer structures (Figure 7d–f), there was a very pronounced overlap of curves for a given amplitude. The presented results of cyclic loading of structures show three characteristic features. The first one is the presence of a hysteresis loop returning almost to zero between each compression cycle.

However, the width of the hysteresis depends on both the type of structure and the amplitude of the cyclic deformation. Another significant feature of the measured curves can be noted, namely the decrease in the value of the compressive load for obtaining a given contraction value in successive cycles with increasing amplitude. This is particularly evident for structures Figure 7a–c,f. One can speak of the Mullins effect in these cases.

By comparing the heights (X2) of the structures before and after mechanical measurements, it was found that they shortened from about 0.3% to 0.5% (residual strain). This indicates that they also underwent a small amount of plastic deformation during compression (the third feature).

## 5. Discussion

The presented results of compression tests for a series of structures made of polymeric re-entrant cells provide an interesting experimental material. First, it can be concluded that the deformation of the structures was reversible during compression and that their cyclic compression resulted in hysteresis curves in the contraction-compression load system. The overlapping loading curves were at the bottom, and the overlapping unloading curves were at the top of the hysteresis loops.

**Figure 8:**
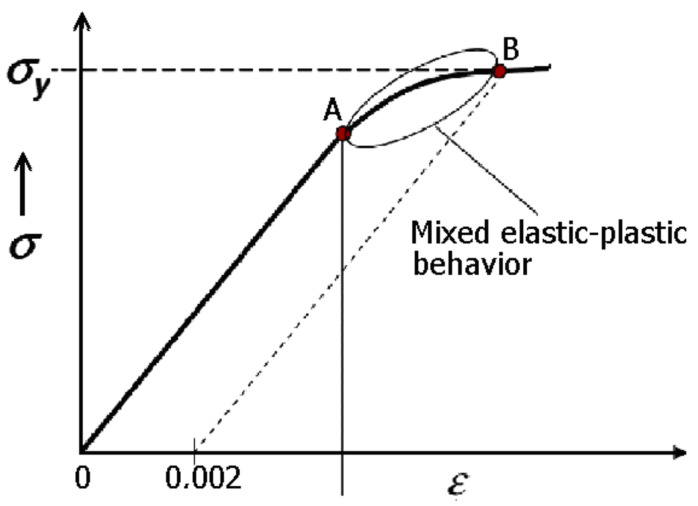
Stress-strain relationship with the linear and nonlinear ranges (where 0-A linear range, A-B non-linear range).

All tested structures demonstrated an elastic behavior, and their compressibility was elastically reversible. It is known that the range of elasticity of materials is limited by the yield strength (B—Figure 8), which usually occurs for deformations greater than that corresponding to the yield point. This range of deformation for metals is schematically demonstrated in Figure 8.

Although the stress increases above the yield point and is no longer linearly proportional to the strain, the material can still behave elastically and return to its original state. Experimental results can be found showing that the stress-strain curve is not necessarily linear in the elastic range, with elastic return and some small residual strain after unloading [22].

Such cases have occurred for the tested structures. In the tested samples, the amount of such permanent residual strain was <0.5%.

A clear difference can be observed between the unloading curve and the reloading curve forming the hysteresis loop, with a repeatable course of unloading (upward convex curve) and loading (downward concave curve)—Figure 7. The width of the hysteresis loop was characteristic of the structures studied and increased along with the increasing amplitude of the cycle. It is assumed that the area of the hysteresis loop corresponds, in general, to the amount of absorbed energy [23].

Other examples of hysteresis loop occurring for auxetic structures under loading are also known, with authors citing various reasons for its presence, i.e., different forms of irreversible deformation. One work [24] reported that the hysteresis loop in cyclic tension-compression tests was due to a rupture in the sample. Also, the hysteresis loop in mechanical testing is found for high compressive stresses [25,26,27].

Another work [28] clearly declared that the presence of hysteresis in the strain-force relationship was the result of plastic deformation. In contrast, a publication on anti-seismic structures [29] emphasized that the stable behavior of hysteresis testifies to its excellent energy absorption capability. Experiments have also shown that the hysteresis for PU foam-filled structures depends on the strain rate, density, and temperature [30,31]. Yet another work [32] obtained hysteresis loops in cyclic compression tests of elastomeric cellular structures.

In an earlier study, the present authors [20] also obtained experimental strain-force curves in the form of hysteresis loops, with the relationship remaining linear for small strains.

In the performed tests, the hysteresis loops for each cycle are repeatable. Overlapping curves were obtained for the five cycles, except for the first compression run. The tested structures exhibited stable behavior in compression and showed no buckling, but only for an amplitude not exceeding about 2/3 of the maximum theoretical strain. For a higher amplitude, the contraction was unevenly distributed along the height, with no cracking.

It is generally known that polymers exhibit a strong time dependence on their strain response to stress and that polymer chains vary in properties in the structure and behavior of the molecules. When the polymer structures tested were subjected to small changes in dimensions (up to 2 mm), the stress-strain response curves obtained were linear and did not show such effects. It can be assumed that the energy barrier for the occurrence of hysteresis is not then exceeded. On the other hand, for higher compression amplitudes, each time a hysteresis response is present. It is assumed that the area under the hysteresis loop corresponds to the energy that is not returned upon unloading but is converted into heat. This phenomenon is characteristic of polymeric materials described as viscoelastic because they contain an elastic part (which stores energy when squeezed) and a viscous part (which converts mechanical energy into heat). The physical explanation for the presence of hysteresis in polymers subjected to cyclic compression includes deformation and disruption of network chains [33]. This involves the loosening of secondary bonds that hold together groups of polymer chains [34].

The experimentally determined hysteresis loops for consecutive compression and relaxation cycles correspond to the well-known Mullins effect [35], which involves stress relaxation and a reduction in compressive stress compared with the stress at the same strain value. In the conducted tests, by increasing the compression amplitude in successive cycles, a decrease in the compressive force for the same value of strain can be observed (Figure 7). This is also referred to as clear stress-softening behavior [33].

The Mullins effect is a particular aspect of the mechanical response of a polymer in which the stress-strain curve depends on the previously encountered maximum load. The Mullins effect, occurring only in elastomers, is known to cause the slight softening of the elastomer during the first few load cycles [35].

**Figure 9:**
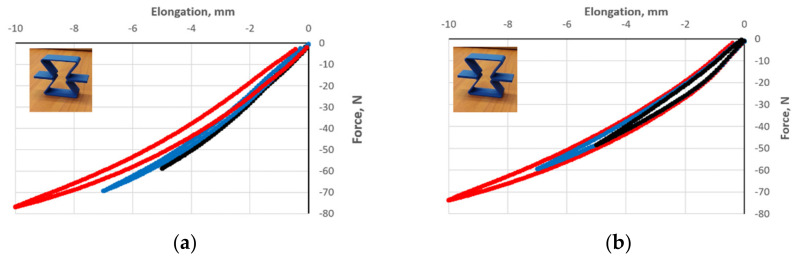
Hysteresis loops for compressing a re-entrant unit cell (t = 2 mm), for increasing amplitude (**a**) and for decreasing amplitude (**b**). The colors indicate the amplitude size: black 5 mm, blue 7 mm, and red 10 mm.

To find the answer to the question of whether the Mullins effect depends on the direction of amplitude change, experiments were performed for the opposite direction, namely for decreasing amplitude values. The results for a single cell are shown in Figure 9. Given the course of the strain-force curves, one can thus also speak of the inverse Mullins effect. Considering, e.g., a deformation of 4 mm, there was a reduction in the value of the needed compressive force—for cycles with increasing amplitude (Figure 9a)—but also for cycles with decreasing amplitude (Figure 9b).

**Figure 10:**
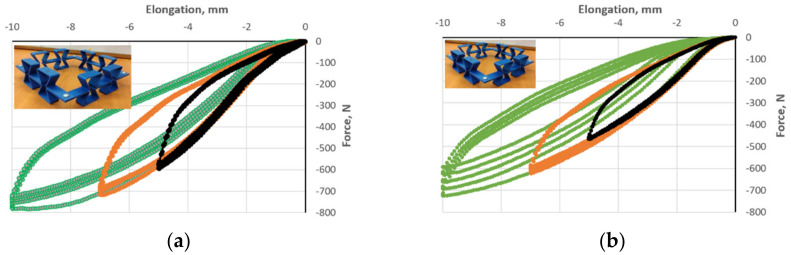
Hysteresis for compressing the re-entrant unit cell structure for increasing amplitude (**a**) and for decreasing amplitude (**b**).

Testing for the occurrence of the Mullins effect for a structure composed of 4 re-entrant unit cells, it was observed that this effect occurs both for the increase (Figure 10a) and for the decrease (Figure 10b) in the cyclic compression amplitude. For a higher compression amplitude, lower values of the force needed to compress the structure to the selected level are obtained.

It should be added that the highlighted occurrence of the Mullins effect for polymeric auxetic structures requires further research and the collection of more extensive experimental material.

Based on the results presented (Figure 9 and Figure 10), it can so far be concluded that the Mullins effect occurs for both individual polymer re-entrant unit cells as well as for polymer re-entrant structures. The Mullins effect does not affect the auxetic behavior of the structures but is only an additional phenomenon related to the unit cell material.

The auxeticity of the studied structures is clearly expressed by the negative Poisson’s ratio (NPR). For the tested structures, the determined values of Poisson’s ratio, calculated from the maximum contraction in compression, approximated the theoretical values.

**Figure 11:**
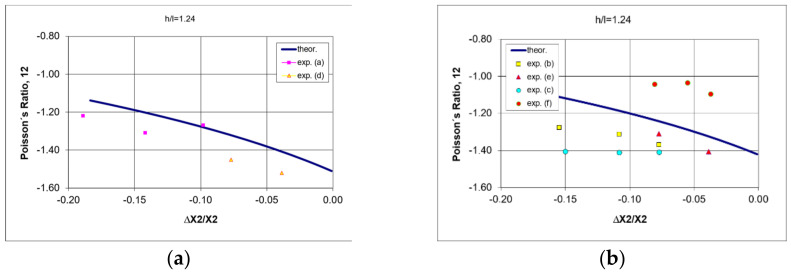
Relationship of Poisson’s ratio and contraction for the tested structures made from re-entrant cells with dimensions (**a**,**b**) h = 46 mm, l = 32.6 mm, θ_0_ = 30° (thickness t = 2.1–2.4 mm, width s = 15 mm, see Figure 2).

Poisson’s ratio decreased to values less than –1 (Figure 11).

The obtained discrepancies between theoretical and experimental values for the produced structures were mainly due to the accuracy of the measurements and the use of theoretical formulae (1) and (3) for the sharp-angled strut joints—as in Figure 11.

The experimental results presented in Figure 11 were determined in the marked measurement series.

Comparison of the theoretical relationships of Poisson’s ratio with experimental quantities shows good agreement, with values below –1 obtained in all cases. This confirms the auxetic nature of these structures, with the largest negative values occurring for very small bending of the structures, which remains in line with the theoretical predictions.

At this point, a question still remains as to the effect of the thickness t of the unit cell struts on the value of Poisson’s ratio.

**Figure 12:**
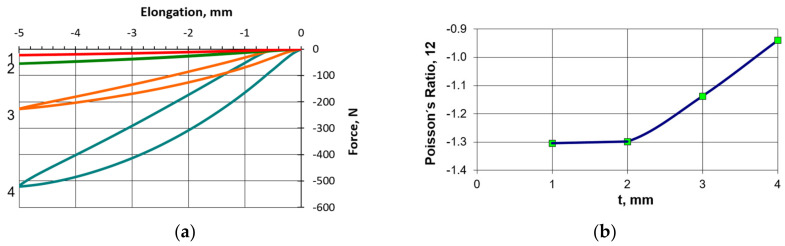
Effect of thickness (t = 1, 2, 3, 4 mm) of struts of an individual unit cell with parameters h = 46 mm, l = 32.6 mm, θ_0_ = 30° on strain-force curves (**a**), and the change in Poisson’s ratio (**b**) with strut thickness t. The different thicknesses of the crossbars are marked in color: red 1 mm, green 2 mm, orange 3 mm and blue 4 mm.

Many theoretical works emphasize the importance of this geometric parameter, which can be easily recognized from the theoretical relationships compiled, for example, in one review work [1]. Given that the rigidity of a material depends on its thickness, the force required to deform it increases with the thickness, though a different behavior may also manifest itself in a thicker material subjected to bending. This was also observed in follow-up studies. The results of additional tests performed for a single unit cell with strut thicknesses of 1, 2, 3, and 4 mm are shown in Figure 12a.

From the results shown (Figure 12a), it can be seen that the compressive force increased with the thickness of the unit cells, while the negative value of Poisson’s ratio for the same values of the degree of deformation of unit cells with different thicknesses decreases significantly (Figure 12b). Such a trend has already been shown in many previous works [1,36], where the Poisson’s ratio value increases for thicker cells.

The performed follow-up study showed that as the thickness of the struts increased, a hysteresis loop appeared in the strain-force relationship (Figure 12a).

## 6. Conclusions

The paper presents the results of studies of re-entrant structures subjected to viscoelastic cyclic compression. The experimental material obtained indicates a number of interesting properties of such structures, which can be regarded as adequately characterizing the behavior of polymeric re-entrant structures when subjected to compression in the elastic range. Already, a single re-entrant unit cell under these conditions shows both hysteresis in the system of stress-strain curves and the Mullins effect for repeated cycles with increasing amplitude. By increasing the thickness of the struts of the re-entrant unit cell, the theoretical relationship between the value of the Poisson’s ratio and strut thickness was confirmed experimentally. It was then shown that for simple structures formed from several re-entrant unit cells, specific changes visible in the stress-strain curves are obtained.

The behavior of several auxetic structures formed by a simple combination of re-entrant unit cells was compared. Each of the combinations studied showed its own peculiarities as to the shape and width of the hysteresis loop. In particular, the results obtained for graded (layered) structures account for their dissimilarity. This indicates that the results of studies done on a single re-entrant unit cell cannot be easily extrapolated onto complex re-entrant structures. It was shown that such auxetic structures exhibit hysteresis in the stress-strain curves and, furthermore, that they confirm the occurrence of the Mullins effect.

Such observations cannot be readily made in widespread simulation studies as they result solely from the design and study of physical metamaterial structures with auxetic properties. The present work confirms the potential of 3D auxetic elastomeric cellular structures for obtaining elastic dimensional changes, which can provide excellent protective properties.

In conclusion, it can be said that the tested structures in compression exhibited auxetic behavior and sustainable deformation properties. The demonstrated spatial structures subjected to compression underwent deformation in the elastic range. This could benefit the creation of more complex structures in the future based on the presented research results, such as in the design of protective systems. Such mechanical metamaterials can become useful not only through rational design and simulation but, more importantly, through their fabrication and testing. This can provide more data and help broaden the knowledge of these engineered mechanical metamaterials.

The elastic behavior of mechanical metamaterial structures enables their integration into various application domains (soft robotics, protective systems) because it introduces advanced functionalities beyond pure mechanics and, above all, expands materials science.

It can be hoped that the observations made in the present work will contribute to a better understanding of auxetic structures and pave the way for future prospects in their manufacturing and application. In future research, the authors will focus on confirming experimentally the auxeticity of more complex spatial re-entrant structures in terms of their applicability.

## Data Availability

The original contributions presented in the study are included in the article; further inquiries can be directed to the corresponding author.
